# Vaccination Coverage Against Human Papillomavirus in Female Students in Cyprus

**DOI:** 10.7759/cureus.28936

**Published:** 2022-09-08

**Authors:** Christothea Konstantinou, Andrew Xanthopoulos, Konstantinos Tsaras, John Skoularigis, Filippos Triposkiadis, Dimitrios Papagiannis

**Affiliations:** 1 Public Health & Vaccines Laboratory, Faculty of Nursing, University of Thessaly, Larissa, GRC; 2 Department of Cardiology, University Hospital of Larissa, Larissa, GRC; 3 Faculty of Nursing, University of Thessaly, Larissa, GRC

**Keywords:** cyprus, coverage, vaccination, hpv, human papillomavirus

## Abstract

Background

Human papillomavirus (HPV) has been associated with the development of several cancers and cardiovascular diseases in females. Nevertheless, there is still poor data on vaccination coverage against HPV in several countries, including Cyprus. The main target of the present research was to assess the vaccination status of female students in Cyprus.

Methodology

An online survey was conducted via a cloud-based short questionnaire on Google Forms. Students with a known email address were initially invited via email to complete the survey. The questionnaire was distributed to 340 students, aged 18-49 years old, who lived in Cyprus (60% response rate).

Results

The total vaccination coverage was 38.1%. The mean age of participants was 23.5 (±6.5) years. The major reason for non-vaccination was the belief that participants were not at risk of serious illness from HPV infection (22%), followed by the reported lack of time to get vaccinated (16%) and inertia (13%). The students who had information about the safety of HPV vaccines from electronic sources of information (television, websites, and blogs) had lower vaccination coverage compared to those who had received information from alternative sources (primary health centers, family doctors, or obstetricians) (relative risk (RR) = 1.923, 95% confidence interval (CI) = 0.9669-3.825; p = 0.033). No significant differences in vaccination rates between participants who were coming from schools of health sciences versus those from financial schools (RR = 1.082, 95% CI = 0.7574-1.544; p = 0.3348) were observed.

Conclusions

Public health policy interventions and education on HPV vaccines are effective ways to improve the awareness and acceptance rate of HPV vaccination among female students and improve the HPV vaccination coverage level in Cyprus.

## Introduction

Worldwide, the fourth most common cancer in women is cervical cancer [1,2]. The human papillomavirus (HPV) and especially strains 16 and 18 are the most frequent gynecological types of cancer, which are associated with 70% of cervical lesions and precancerous cervical wounds [3,4]. According to the World Health Organization (WHO), in 2020, cervical cancer was globally responsible for 604,000 new cases and 342,000 deaths [1,2]. In addition, HPV has been associated with the development of cardiovascular disease (CVD), especially in females [5-7]. Therefore, vaccination against HPV is of utmost importance [2].

Several vaccine programs for women have been adopted in many countries since June 2006 [8,9]. An increasing number of countries expand the indications and include males aged 9-26 years in vaccination programs [8]. A total of 68 countries and 12 regions have been included in the vaccination program against HPV until October 2014 [10]. The ultimate goal of the vaccine against HPV was to administer two doses to females under 15 years of age [11], whereas 118 million women globally have joined the vaccination program [10].

The WHO suggests that national immunization programs on HPV vaccination must be implemented for females aged 9-14 years (primary target population) and females aged ≥15 years or adolescent males (secondary target population) [12]. Some countries that implement national vaccination programs have achieved better vaccination coverage in preadolescent girls (9-14 years old) compared to countries where vaccination against HPV is given at the demand of primary healthcare services (family doctors, obstetricians, and nurses) [4,13]. Cyprus has developed and advanced a national vaccination program, which ensures excellent protective capabilities in public health. The Ministry of Health and the Pediatric Society of Cyprus recommend the administration of the HPV vaccine at the age of 11 to 12 years. In Cyprus, the vaccine is provided free of charge to girls aged 11-13 years [14]. The most recently available data about HPV vaccination coverage is for the period 2016-2020. The percentage of 15-year-old girls who were fully vaccinated was 54% in 2018, 59% in 2019, and 64% in 2020 [15].

To our knowledge, there are still limited data about vaccination coverage of female students regarding vaccination against HPV in Cyprus. The aim of this study was to assess the vaccination status of female students in Cyprus and possible associated factors, to record the proportion of participants who have received the first dose of the HPV vaccine, and to record the proportion of those who have completed the two or three-dose series.

## Materials and methods

An online survey was conducted via a cloud-based short questionnaire disseminated on Google Forms. During the coronavirus disease 2019 (COVID-19) pandemic, several quantitative studies have been conducted worldwide adopting this technique, making it popular and proving its effectiveness on speed and range, convenience, cost, flexibility, and automation [16,17]. Students from three schools were invited to participate in the anonymous online survey, and 39 medicine students out of 208 (18.5%) total students participated; 78 (37.7%) students from biology, and 91 (43.8%) students from the school of economics of the Universities of Cyprus. Students with a known email address were initially invited via email to complete the survey. The questionnaire was distributed to 340 students, aged 18-49 years, who lived in Cyprus (60% response rate). Each prospective participant was informed initially about the aims and procedures of the study and could then freely choose whether to participate. Students were allowed to leave the interview midway if they felt uncomfortable. All study participants provided consent.

The study protocol was approved by the Scientific Committee for the Medical Laboratories Under Graduate program of the University of Thessaly, Greece (8891/ΣΕ/20-7-2021).

The questionnaire included items about demographics (age, nationality, school) and two questions on the student’s attitudes toward the importance of vaccination regarding public health protection (five-point Likert scale: fully agree, agree, uncertain, disagree, completely disagree). The same questionnaire was used by Papagiannis et al. in an older study conducted in Greece [4]. Participants were asked to express their general opinion about vaccination (agree, fully agree, uncertain, disagree, fully disagree). In addition, participants were asked “Have you been vaccinated against Human papillomavirus (HPV)?” (Yes/No). If they chose “Yes,” the next (forward) question was “How many doses?” (one, two, or three). If not, participants were asked to specify why they did not want to get vaccinated. Participants were asked to evaluate their knowledge about the safety of the HPV vaccine (insufficient, sufficient, no information, excellent, uncertain). Moreover, they were required to answer the question “Do you have any information about sexual diseases caused by HPV?” (Yes/No). They had to choose which symptoms they considered to be caused by HPV infection based on their own judgment. The options included genital warts, cervical cancer, vaginal cancer, vulvar cancer, and penile cancer. Finally, students were requested to report the source of information about the safety of HPV vaccines. The information source choices were family doctors, primary health centers (nurse, midwife), TV/website/blogs, magazines/newspapers, and obstetricians.

The completed questionnaires were imported into Microsoft Excel 2016, and the collected data were restored into a database. The Open Epi Info Software version 3.01 was used to calculate the epidemiologic statistics [18]. Absolute (n) and relative frequencies (%) were presented for qualitative variables, while the mean (±standard deviation (SD)) was used for the presentation of continuous variables. The chi-square test was used for the univariate analysis of qualitative variables, and the Student’s t-test was used for the univariate analysis of continuous data. The relative risk (RR) and 95% confidence intervals (CIs) were calculated. The level of statistical significance was set at 0.05.

## Results

The number of participants (who answered the questions) in the present study was 208. Their mean age was 23.5 (±6.5 SD) years. Overall, 43.8% of the participants were students from the school of economics, 37.7% were biology students, and 18.5% were medical students. The majority of participants were Cypriots (62.5%), followed by Greeks (33%), South Africans (2%), Kenyans (1.5%), and Armenians (1%). Most of the participants (73%) reported that immunizations are an important tool to protect public health. In total, 79 of the participants reported that they had been immunized for HPV (two or three shots of vaccine). Moreover, 29 of the vaccinated participants had been given two shots of the vaccine and 50 had been given three shots. However, the majority of subjects had not received the vaccine. The major reason for not getting vaccinated was the belief that female students were not at risk of serious illness from HPV infection (22%), followed by the reported lack of time to get vaccinated (16%) and inertia (13%) (Table 1). Interestingly, 12% of participants expressed their objections to the safety of the HPV vaccine. The majority reported that they had been informed about sexually transmitted diseases (STDs) caused by HPV (72%). The most common STD symptom caused by the HPV infection was reported to be genital warts (69%), followed by cervical cancer (66%), vulvar cancer (36%), vaginal cancer (25%), and penile cancer (16%).

**Table 1:** Characteristics of female students and their attitudes toward HPV vaccination. SD: standard deviation; STD: sexually transmitted disease; HPV: human papillomavirus

Characteristics	n/total (%) or mean (±SD)	
Sex/Female	208	100%
Age	23.5 ± 6.5	
Nationality		
Cypriot	130/208	62.5%
Greek	69/208	33%
South African	4/208	2%
Kenyan	3/208	1.5%
Armenian	2/208	1%
School of science training		
School of economics	91/208	43.8%
Biologist	78/208	37.7%
Medical school	39/208	18.5%
Vaccinations are important for protecting public health		
Agree	52/208	25%
Fully agree	99/208	47.5%
Uncertain	41/208	19.7%
Fully disagree	9/208	4.3%
Disagree	7/208	3.5%
My opinion on vaccination is		
Agree	64/208	31%
Fully agree	85/208	41%
Uncertain	41/208	20%
Fully disagree	9/208	4.5%
Disagree	8/208	3.5%
Have you been vaccinated with the HPV vaccine?		
Yes	79/208	38%
No	129/208	62%
If yes how many doses		
Three doses	50/79	63%
Two doses	20/79	37%
If no, please specify		
I don’t have enough time	34/208	16%
Inertia	28/208	13%
Use of alternative drugs	3/208	1%
I am not at risk of serious illness	45/208	22%
Fear over vaccine safety	25/208	12%
Do you have information about STDs caused by HPV?		
Yes	151/208	72.5%
No	57/208	27.5%
The most common STDs caused by HPV is		
Penile cancer	35/208	17%
Vaginal cancer	52/208	25%
Vulvar cancer	74/208	36%
Cervical cancer	138/208	66%
Genital warts	144/208	69%
From the risk factors of HPV, the most important is		
Number of sex partners	82/208	39%
Personal contact	22/208	11%
Skin damages	5/208	2%
Age to start the sexual activity	3/208	1%
All	98/208	47%

In univariate analysis, no significant differences in vaccination rates between participants who were coming from schools of health sciences versus those from the school of economics (RR = 1.082, CI = 0.7574-1.544; p = 0.3348) were observed. Finally, the majority of participants (49%) answered that the number of sex partners was the most important risk factor for HPV infection (Table 1). As expected, students who had a positive opinion about vaccinations exhibited a significantly higher probability to get vaccinated against HPV compared to those who disagreed (RR = 14.89, CI = 3.781-58.62; p < 0.0000001). The students who had a sufficient opinion about STDs caused by HPV were documented to have a double proportion of vaccination against HPV in comparison with those who were not sufficiently informed about the STDs caused by HPV (RR = 1.916, 95% CI = 1.15-3.194; p = 0.0026). Students who had been informed by electronic sources had lower vaccination coverage. In comparison, students who had received information on HPV vaccine safety from other sources (primary health centers, family doctors, or obstetricians) had higher vaccination coverage for the HPV vaccine (RR = 1.923, CI = 0.9669-3.825; p = 0.0336) (Table 2).

**Table 2:** Factors influencing HPV vaccination uptake. RR: relative risk; CI: confidence interval; STD: sexually transmitted disease; HPV: human papillomavirus

Variable	HPV		
	n/total (%)	RR (95% CI)	P-value
School of Health Sciences	46/116 (39%)		
School of Economics	33/92 (36%)	1.082 (0.7574-1.544)	0.3348
My general opinion about vaccinations is			
Agree	77/150 (51%)	14.89 (3.781-58.62)	<0.0001
Disagree/Uncertain	2/58 (3.4%)		
Do you have information about STDs caused by HPV?			
Yes	66/79 (83.5%)	1.916 (1.15-3.194)	0.0026
No	13/79 (16.4%)		
Mass media information on HPV vaccination			
Yes	13/79 (16.4%)	1.923 (0.9669-3.825)	0.0336
No	66/79 (83.5%)		

## Discussion

HPV is one of the most common sexually transmitted infections worldwide. It has been associated with cancer and CVD, especially in women [3-7]. Currently, there are limited data on HPV vaccination coverage in Cyprus. In this study, we report results of the vaccination coverage in female students in Cyprus according to the national HPV vaccination program. Since the implementation of this program, 38% of female participants have been fully vaccinated for HPV.

According to the European Centre for Disease Prevention and Control (ECDC), in May 2012, the vaccination advisory bodies in 12 of the 29 countries made a recommendation in favor of HPV vaccination compared to 12 out of 27 countries in February 2008. In contrast Cyprus, Estonia, Finland, Hungary, Lithuania, Malta, Poland, and Slovakia have not introduced since 2012 a national immunization program, nor have their vaccination advisory boards made recommendations about the HPV vaccination [19].

Cyprus introduced the HPV vaccine in the National Immunization Program in 2016 [20]. There are currently three HPV vaccines licensed in Europe including Cyprus. The bivalent vaccine contains virus-like particles of HPV strains 16 and 18; the quadrivalent HPV vaccine includes HPV strains 6, 11, 16, and 18; and the new nine-valent vaccine contains HPV strains 6, 11, 16, 18, 31, 33, 45, 52, and 58 [21]. Following the first introduction of the HPV vaccines in 2006, ECDC provided guidance on how to identify target populations for HPV vaccination, support the identification of strategy options for HPV vaccine delivery in European Union (EU) countries, model costs and outcomes of HPV vaccination, and monitor and evaluate the impact of HPV vaccination [13].

Our study presents slightly different results compared with a recent study conducted at the University of Nicosia, which reported that 30.7% of the participating students had been vaccinated against HPV vaccine [22]. The total HPV vaccination coverage in this study was 38%, which was low despite the fact that the HPV vaccine was introduced relatively early by the Cyprus National Immunization Program [14]. Different vaccination rates (44.3%) have been recorded in older studies in female students of health professions in Greece (two or three doses of vaccine against HPV) [4] in comparison with a study by Donadiki et al., where only 25.8% of high education students reported that they had received three doses of the HPV vaccine [23].

In this study, students who had a positive opinion about vaccinations exhibited increased vaccination coverage rates against HPV in comparison to the students who were against vaccinations. Observed positive association between the reported vaccination rate and knowledge level, as shown in the current and previous studies [4,23-25], highlights the need for educational activities in students. A recent study conducted in China, a country with a large number of new cases of HPV infections [26], attempted to investigate HPV vaccination rates, knowledge, acceptability, and associated factors in college students and revealed that 36.9% of females and 24.8% of males would choose to get HPV vaccination [27]. In line with previous findings, the vast majority of students in this research reported that HPV vaccines are safe and effective and generally agreed with the vaccination policies [4].

In this study, the proportion of immunization against HPV vaccine was lower in female students who had been informed about the HPV vaccine safety by mass media compared to students who had been informed by other sources such (primary health centers, family doctors, or obstetricians). According to the published literature, a major factor associated with vaccination acceptance is the recommendation for vaccination from physicians and healthcare professionals [4,27,28].

In this study, one out of five participants reported that the major reason for not getting vaccinated was the belief that they were not at risk of serious illness from HPV infection. Many published studies have discussed the beliefs and philosophical reasons that drive people to delay vaccination, especially with regards to new vaccines, although vaccination is considered to be one of the greatest achievements of public health [29,30]. In the recent pandemic, several studies have indicated that the perceived low COVID-19-related health risks are a common reason for under-vaccination.

Another finding of the present study was that 12% of subjects reported being reluctant to get the HPV vaccine due to safety concerns. Usually, fear of side effects is a common barrier against parental acceptance of HPV vaccination and has been reported in previous studies [4,31,32].

The HPV vaccines confer some degree of cross-protection against non-vaccine HPV serotype infection and precancerous cervical lesions. There are still concerns about the duration of protection of vaccination beyond nine years based on vaccine effectiveness and the possibility of a booster dose [33,34].

Strategies to increase public participation in HPV vaccination should be implemented. For example, vaccines may be available in schools and pharmacies [35]. In countries such as Australia, the United Kingdom, and Portugal, which had school-based interventional programs, women received more than 80% of the vaccine [36].

There are several limitations of this study that need to be acknowledged. Circumstantial analysis of the individual-level factors associated with vaccination uptake was challenging as the data were collected using a self-reported questionnaire. Human error may occur in data collection. Moreover, our results may not be fully applicable to the entire Cypriot population.

## Conclusions

In our sample, we report low vaccination coverage for female students in Cyprus against the HPV vaccine. Several interventions can be implemented to increase the acceptance of the HPV vaccine among female students. Our data were collected from a small sample of students in Cyprus and cannot be generalized to the entire population. Public health policy interventions and education on HPV vaccines are effective ways to improve the awareness and acceptance rate of HPV vaccination among female students, thus improving the HPV vaccination coverage level in Cyprus.
